# Real life data: follow-up assessment on Spanish Gaucher disease patients treated with eliglustat. TRAZELGA project

**DOI:** 10.1186/s13023-023-02939-4

**Published:** 2023-12-15

**Authors:** Irene Serrano-Gonzalo, Laura López de Frutos, Carlos Lahoz-Gil, Francisco Delgado-Mateos, María Ángeles Fernández-Galán, Montserrat Morales-Conejo, María Victoria Calle-Gordo, Daiana Ibarretxe-Gerediaga, Andrés Madinaveitia-Ochoa, Antonio Albarracin-Arraigosa, José Balanzat-Muñoz, Patricia Correcher-Medina, Luis Javier García-Frade, Jesús María Hernández-Rivas, Francesca Labbadia, Jesus Miguel López-Dupla, María Luisa Lozano-Almela, Elvira Mora-Casterá, María Soledad Noya-Pereira, María Ángeles Ruíz-Guinaldo, María del Mar Tormo-Díaz, Isidro Vitoria-Miñana, Isidro Arévalo-Vargas, Marcio Andrade-Campos, Pilar Giraldo

**Affiliations:** 1 Fundación Española para el Estudio y Terapéutica de la Enfermedad de Gaucher y otras lisosomales (FEETEG), Saragossa, Spain; 2 Grupo de Investigación de Enfermedad de Gaucher (GIIS-012), Instituto de Investigación Sanitaria de Aragón, Saragossa, Spain; 3 Grupo Español de Enfermedades de Depósito Lisosomal (GEEDL), Sociedad Española de Hematología y Hemoterapia, Saragossa, Spain; 4 Servicio de Hematología, Hospital Punta de Europa, Cádiz, Spain; 5 Servicio de Hematología, Hospital Virgen del Puerto, Plasencia, Spain; 6 Servicio de Medicina Interna, Hospital Universitario, 12 de Octubre, Madrid, Spain; 7 Servicio de Hematología, Hospital de Torrecardenas, Almería, Spain; 8 Unitat de Medicina Vascular i Metabolisme (UVASMET), Unitat de Recerca en Lípids i Arteriosclerosis, Hospital Universitari San Joan, CIBER de Diabetes y Enfermedades Metabólicas Asociadas, Instituto de Salud Carlos III, IISPV, Universitat Rovira i Virgili, Reus, Spain; 9 Servicio de Hematología, Hospital Comarcal de Alcañiz, Teruel, Spain; 10 Servicio de Medicina Interna, Hospital Serranía de Ronda, Málaga, Spain; 11 Servicio de Hematología, Hospital Can Misses, Ibiza, Spain; 12 Unidad de Nutrición y Metabolopatías, Hospital Universitari i Politècnic La Fe, Valencia, Spain; 13 Servicio de Hematología, Hospital Universitario Río Hortega, Valladolid, Spain; 14 Departamento de Medicina, Universidad de Salamanca and Servicio de Hematología, Hospital Universitario de Salamanca, Salamanca, Spain; 15 Servicio de Hematología, Hospital de La Vega Lorenzo Guirao, Murcia, Spain; 16 Servicio de Medicina Interna, Hospital Universitari Joan XXIII, Tarragona, Spain; 17 Servicio de Hematología, Hospital General Universitario Morales Meseguer, IMIB-Pascual Parrilla, CIBERER-U765, Murcia, Spain; 18 Servicio de Hematología, Hospital Universitari i Politècnic La Fe, Valencia, Spain; 19 Servicio de Hematología, Hospital Teresa Herrera, A Coruña, Spain; 20 Servicio de Hematología, Hospital Comarcal Francesc de Borja, Valencia, Spain; 21 Servicio de Hematología, Hospital Clínico Universitario de Valencia, Valencia, Spain; 22 Servicio de Hematología, Hospital QuirónSalud, Saragossa, Spain

**Keywords:** Type 1 Gaucher disease, Biomarkers, Glucosylsphingosine, Lipocalin-2, YKL-40, SF-36, Eliglustat

## Abstract

**Background:**

The availability of multiple treatments for type 1 Gaucher disease increases the need for real-life studies to evaluate treatment efficacy and safety and provide clinicians with more information to choose the best personalized therapy for their patients.

**Aims:**

To determine whether treatment with eliglustat produces, in adult GD1 patients, ans optimal response in daily clinical practice.

**Methods:**

We designed a real-life study with 2 years of follow-up (TRAZELGA [GEE-ELI-2017-01]) to uniformly evaluate the response and adverse events to eliglustat treatment. This study, conducted in 30 patients across Spain and previously treated with other therapies, included the evaluation of safety and efficacy by assessing visceral enlargement, bone disease (DEXA and T and Z scores), concomitant treatments and adverse events, as well as a quality of life evaluation (SF-36). In addition, the quantification of classical biomarkers (chitotriosidase activity, CCL18/PARC and glucosylsphingosine (GluSph)) and new candidates for GD biomarkers (YKL-40, cathepsin S, hepcidin and lipocalin-2 determined by immunoassay) were also assessed. Non-parametric statistical analysis was performed and *p* < 0.05 was considered statistically significant.

**Main Results:**

Thirty patients were enrolled in the study. The median age was 41.5 years and the male–female ratio was 1.1:1. 84% of the patients had received ERT and 16% SRT as previous treatment. The most common symptoms at baseline were fatigue (42%) and bone pain (38%), no patient had a bone crisis during the study, and two years after switching, 37% had reduced their use of analgesics. Patient-reported outcomes showed a significant increase in physical function scores (*p* = 0.027) and physical pain scores (*p* = 0.010). None of the enrolled patients discontinued treatment due to adverse events, which were mild and transient in nature, mainly gastrointestinal and skin dryness. None of the biomarkers show a significant increase or decompensation after switching. CCL18/PARC (*p* = 0.0012), YKL-40 (*p* = 0.00004) and lipocalin-2 (*p* = 0.0155) improved after two years and GluSph after one year (*p* = 0.0008) and two years (*p* = 0.0245) of oral therapy.

**Conclusion:**

In summary, this real-life study, showed that eliglustat maintains stability and can improve quality of life with few side effects. Significant reductions in classic and other novel biomarkers were observed after two years of therapy.

**Supplementary Information:**

The online version contains supplementary material available at 10.1186/s13023-023-02939-4.

## Introduction

Gaucher disease (GD) (MIM#230800; #230900; #231000) is an autosomal recessive inherited disorder caused by a functional defect in the enzyme acid glucocerebrosidase (GluCer) (EC3.2.1.45) induced by pathogenic variants in the *GBA1* gene (MIM*606463). This defect leads to an accumulation of glucocerebroside, mainly in the lysosomes of macrophages, and causes deterioration of the organs and tissues in which it is stored. The accumulation of undegraded substrate induces a multisystemic disease of variable expression in terms of clinical manifestations, course and severity [1]. The disease is classified into three forms according to neurological involvement, with the most common form being type 1 Gaucher disease (GD1; MIM#230800) [2].

The clinical spectrum of GD1 is highly variable, ranging from mild phenotypes with no clinical manifestations to others with severe symptoms; among the complications, bone disease is the most disabling and painful. In addition, GD is characterized by a latent state of chronic inflammation secondary to macrophage activation, expressed by an imbalance in proinflammatory cytokine production, hyperferritinaemia, hypergammaglobulinaemia, impaired calcium homeostasis and metabolic syndrome, which are likely to influence the development of disease complications [3, 4].

Treatment of the disease involves two different modes of action. Enzyme replacement therapy (ERT) was the first to be approved, with two different drugs available in Spain (imiglucerase [5]. and velaglucerase alfa [6]), both administered intravenously every 15 days. The second mode of action is substrate reduction therapy (SRT), also with two different drugs (miglustat [7]. and eliglustat [8]), which are administered orally.

ERT has been widely used for more than 25 years to treat both type 1 and type 3 patients. The therapy is usually well tolerated and considered safe, but there are aspects not covered by the treatment, such as neurological involvement and, in some patients, skeletal complications [9, 10]. Eliglustat (Cerdelga®, Sanofi Genzyme, Cambridge, MA, USA) is the last approved treatment, available in Spain since 2015 for the treatment of adult GD1 patients as first or second line therapy [8]. This drug inhibits the enzyme glucosylceramide synthase (EC2.4.1.80) by reducing glucosylceramide synthesis and thus intralysosomal accumulation. Eliglustat is primarily metabolized by the cytochrome CYP2D6 pathway and is not available to patients with ultra-rapid metabolism (10%) [11]. Results from clinical trials have shown that eliglustat, as first-line therapy or when switching from an ERT, provides an optimal response with improvement or stabilization of clinical features [12–14]. Following approval of the drug, several "real-world" studies have been conducted to determine safety and efficacy outside the clinical trial setting, with similar results [15–18].

Several biomarkers have been used to screen for suspected disease and to monitor treatment compliance [19]. Chitotriosidase activity (ChT) [20], cytokine CCL18/PARC [21] and glucosylsphingosine (GluSph) [22] levels were found to be elevated in patients with GD at diagnosis compared to healthy controls and decreased during treatment. Other potential biomarkers, such as chitinase YKL-40, have been found to be elevated in cellular and animal models of GD [23]. In addition, molecules involved in the inflammatory milieu have been identified by gene array in the spleen of GD mice as potential biomarkers, including lipocalin-2 and cathepsin S [24]. Subsequent studies have shown statistically significant differences between controls and GD patients for both YKL-40 and cathepsin S, and in the case of cathepsin S also between untreated and ERT-treated patients [25].

The regulation of iron metabolism is limited to the inhibition of iron absorption in the intestine by hepcidin, a peptide produced by hepatocytes whose production is influenced by inflammatory cytokines [26]. Significant differences in hepcidin concentration have been observed in GD patients treated with miglustat compared to untreated patients, while no differences were found between GD patients and healthy controls [27].

The main objective of this project was to determine whether treatment with eliglustat in adult GD1 patients provides an optimal response in daily clinical practice. Furthermore, whether this response is reflected in plasmatic biomarkers and patients' quality of life.

## Methods

### Patients

The study was performed between May 2019- September 2022 and included adult patients with GD1 from all over Spain who were being treated with eliglustat. Patients were enrolled at the discretion of their physician (in accordance with the indications specified in the SPC of the drug) [8] and on the basis of their own willingness to participate. All participants signed a study specific informed consent, which was approved by the Research Ethics Committee of the Community of Aragón (Spain). The inclusion/exclusion criteria are detailed in Additional file 1: Table S1.

In addition all patients, previously to start therapy with eliglustat are evaluated by a cardiologist and perform an ECG and echocardiogram, it is a recommendation in Spanish patients before prescription.

### Demographic, clinical and laboratory data

*GBA1* genotype, clinical data, liver and spleen volumes, blood counts, biochemical and hemostatic parameters were collected before the switch to eliglustat treatment and after 1 and 2 years on this therapy and were provided by the physicians, as well as bone density assessment (DEXA). T- and Z-scores were analyzed taking into account the age and sex of the patients, as previously reported [28].

Magnetic resonance imaging (MRI) was analyzed by the same expert before the treatment change and one and two years later. S-MRI was calculated as previously published [29].

The CYP2D6 metabolizer profile was assessed in all patients prior to switching, as previously published [30]. In addition, the CYP2D6 activity score was calculated according to the recommendations of Gaedigk et al. [31].

### Adverse events and patient-reported outcomes

The presence of adverse events during the study was reported by the patients' physicians in a form filled out at the same time that the biological sample was collected.

Patient-reported outcomes (PROs) were assessed using the Short Form Health Survey quality of life questionnaire before starting eliglustat treatment and after the first and second year of treatment. This information was compared with general Spanish population data published [32].

All patients were inquiry about the intake of analgesic and NSAIDs in order to have indirect information about the pain level.

### Biomarkers

The panel of biomarkers was evaluated at baseline and every 6 months during therapy.

ChT activity was measured by fluorometry using 4-methylumbelliferone-b-D-triacetylchitotriosidase as substrate (Sigma Chemical Co., St. Louise, MO, USA), as previously described [33]. Concentrations of the cytokines CCL18/PARC, YKL-40, cathepsin S, hepcidin and lipocalin-2 were analyzed by immunoassay according to the manufacturer's instructions (DY394, DY2599, DY1183, DY8307 and DY1757, respectively, R&D Systems Europe, Ltd). Finally, GluSph concentration was measured by LC–MS/MS as previously published [34].

### Statistics

Statistical analyses were performed using R software (version R-4.2.0). Descriptive analysis was performed with median and interquartile range for quantitative variables and percentages and frequencies for qualitative variables.

The non-parametric Wilcoxon test was used to assess differences for biomarkers before eliglustat treatment and at one and two years.

For PROs, means were compared by t-test between baseline and two years.

Differences with *p* value < 0.05 were considered statistically significant.

## Results

### Patients included

Thirty patients were enrolled in the study. None of the patients were naïve, and most were switched to eliglustat after several years of ERT (84%). All patients had completed 24 months of eliglustat therapy. The median age of the patients was 41.5 (Q1–Q3: 30.50–55.00) years, and the gender distribution was 47% female and 53% male. *GBA1* genotype, median years on therapy prior to eliglustat and prior therapies are detailed in Table 1.

**Table 1:** Demographic data

Variable	Males (n = 16)	Females (n = 14)	Total (n = 30)
Median age years (Q1–Q3)	42.5 (28.25–58.00)	41.5 (31.00–53.50)	41.5 (30.50–55.00)
*GBA1* genotype (NM_000157)			
c.1226A > G + c.1226A > G (%)	3	1	4 (13%)
c.1226A > G + c.1448T > C (%)	5	7	12 (40%)
c.1226A > G + other (%)	6	5	11 (37%)
Other + Other (%)	2	1	3 (10%)
Previous spleen removal (%)	1	3	4 (13%)
Median years on previous therapy (Q1–Q3)	17.0 (7.00–22.00)	16.0 (12.75–21.00)	17.0 (8.00–20.75)
Previous treatment*			
Imiglucerase (%)	6	7	13 (43%)
Velaglucerase alfa (%)	8	4	12 (40%)
Miglustat (%)	3	3	6 (20%)
*Comorbidities:*			
Arterial hypertension (%)	3	1	4 (13%)
Diabetes (%)	2	0	2 (6%)
Previous neoplasias (%)	0	3	3 (9%)
*CYP2D6* Activity Score (AS)			
AS = 0 (poor metabolizer)			1 (3%)
AS = 0.5 (intermediate metabolizer)			4 (13%)
AS = 1 (extensive metabolizer)			6 (20%)
AS = 1.5 (extensive metabolizer)			6 (20%)
AS = 2 (extensive metabolizer)			13 (44%)

Information about the age, treatments, genotypes and comorbidities from all patients

All this information was obtained before start eliglustat treatment. The % was calculated only for the total of patients. AS: activity score

*One patient received two treatments simultaneously: imiglucerase and miglustat

### Clinical data

#### Clinical symptoms

Pain information was reported by 13 patients before switching and after two years of treatment with eliglustat. Before the switch, 5 patients (38%) reported pain, two of them diffuse and three localized. After two years of treatment, 5 patients also reported pain, but two of them did not before the switch, both reporting localized pain. In addition, two patients did not report pain after the switch, one with diffuse and one with localized pain before the switch.

Information about fatigue was reported by 12 patients, 5 of them (42%) reported this symptom before the switch and 4 of them did so after 2 years of treatment (33%). Two patients reported an improvement of this symptom with the new treatment, while one patient reported a worsening of the symptom.

Eight patients reported intake additional drugs as analgesics, NSAIDs, antidiabetic, antihypertensive or antidepressants. A 37% reduction in analgesic drug use two years after switching were reported in these patients.

At the time of inclusion, 23% of patients had comorbidities such as diabetes, arterial hypertension or cardiovascular event, previous neoplasia.

#### Visceral involvement

Four of the patients underwent splenectomy prior to conversion (13%) and were excluded from the assessment of splenomegaly. Of the remaining patients, only 17 reported information on the spleen, with mild enlargement in 7 patients (41%) before the switch and maintained in the same patients after one and two years of treatment. 15 patients reported information on liver size, only one of them had a mild hepatomegaly that was reduced after two years of treatment.

In Table 2 we have detailed the clinical manifestations collected by the physicians during the baseline and follow-up visits.

**Table 2:** Clinical characteristics of patients including in the study and follow-up

Clinical manifestations	Baseline (n = 29)	After 12 months (n = 28)	After 24 months (n = 28)	*p* value
Pain (%)	12/29 (47.7)	10/28 (37.7)	9/28 (32.1)	0.4699
Fatigue (%)	7/26 (26.9)	5/26 (19.2)	5/26 (19.2)	0.6145
Spleen enlargement* (range in cm)	10/23 (12–22)	10/24 (12–20)	5/16 (12–20)	–

*4 patients previously splenectomized were not included. The spleen exam was performed by ultrasounds and only consider enlargement values up 12 cm. In bracket the ranges

#### Bone disease

In terms of global bone involvement, the mean S-MRI at baseline was 5.2 (Q1–Q3: 3.34–7.08), after 1 year of therapy it was 4.7 (Q1–Q3: 2.86–6.45) (*p* = 0.008316) and after 2 years of eliglustat therapy it decreased to 4.2 (Q1–Q3: 2.57–6.05) (*p* = 0.003434). It should be noted that more than 50% of patients had residual bone complications such as avascular necrosis, infarcts, joint replacement that are not reversible.

Eight patients report information on bone mineral density assessment before switching and after two years of treatment. One patient (13%) recovered from osteoporotic status to normal, while two (25%) deteriorated from normal status to osteopenia. 5 patients (62%) maintained their DEXA values without changes since the switch.

T and Z values at the lumbar spine were reported by 7 patients before the switch and after two years. Three of them met the evaluation criteria for T-score, with values of -1.3 (− 1.86/− 0.62) before the switch and − 2.2 (− 2.45/− 1.95) two years after the switch. The other 5 patients had Z-scores in the same range, with median and interquartile range values of − 1.6 (− 1.95/− 1.55) and − 0.5 (− 1.40/− 0.25) before and after 2 years, respectively. The differences were not statistically significant (*p* = 0.180 and *p* = 0.285, respectively).

### Adverse events and patient reported outcomes

#### Adverse events

During the first year of treatment with eliglustat, patients generally reported good tolerability with mild and transient adverse events. After two years of treatment with eliglustat, only 26 patients reported adverse events. Grade 1 dyspepsia was reported by 27% (7/26) of patients, dry skin by 11% (3/26), and anxiety, hallucinations, headache, and constipation by 4% (1/26) each. One patient had a *Helycobacter pilory* infection.

#### COVID-19

As the study was conducted during the COVID-19 pandemic, physicians were asked for information on whether any patients had developed severe symptoms of COVID-19 or undergone changes in GD treatment, during lockdown in Spain. During the first 12 months, none of the patients experienced symptoms of COVID-19 or a change in treatment regimen. After two years, most patients had adhered to the established vaccination schedule and 7 patients reported mild coronavirus infection.

#### Patient-reported outcomes

**Fig. 1:**
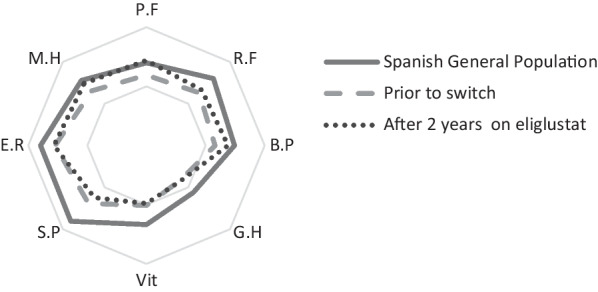
Results of SF-36 enquiry. Radial plot of all categories of the SF-36 questionnaire and global punctuation for the healthy population (data previously published by Alonso et al. [30]) and for our study population before switching and after 2 years with the new treatment. P.F: physical functioning; R.F: physical role functioning; B.P.: bodily pain; G.H.: general health perception; Vit: vitality; S.P.: social role functioning; E.R.: emotional role functioning; M.H.: mental health

At baseline, both physical and mental health domains showed significant differences between GD patients compared to the Spanish general population. The main differences were observed in physical functioning, bodily pain, general health, vitality and emotional role. The comparative study was conducted between baseline and after two years of eliglustat therapy. A significant increase in physical function (*p* = 0.027), bodily pain (*p* = 0.010) and a trend in mental health (*p* = 0.067) was observed in this cohort after two years of eliglustat therapy compared to baseline (Fig. 1).

### Analytical tests

Hematological and biochemical information was collected before the treatment switch, at 12 months, and at 24 months on the new therapy.

Most of the values were in the normal range before starting eliglustat, with the exception of glucose and ferritin, both of which increased when compared to normal ranges for the adult population. The mean of the collected analytical data remained stable after two years of therapy.

Ferritin was higher than reference values, with a greater increase in men than in women. After 24 months of eliglustat treatment, levels were close to the normal range, with a notable decrease in men. (Table 3). Protein profile data showed two patients with gammopathies at baseline (7%), one monoclonal and the other polyclonal.

**Table 3:** Analytical results

Variable (normal values)	At switch (n = 28)	12 months (n = 27)	24 months (n = 21)
Hemoglobin (11.5–16 g/dL)	13.9 ± 1.6	13.9 ± 1.8	13.5 ± 1.6
Hematocrit (36–51%)	41.5 ± 2.7	41.6 ± 2.9	40.5 ± 2.8
VCM (80.0–101.0 fl)	90.1 ± 6.0	91.5 ± 5.2	92.0 ± 4.1
WBC (4.5–11 × 10^9^/L)	7.3 ± 3.5	6.2 ± 1.6	6.5 ± 2.3
Neutrophils (2.6–8.5 × 10^9^/L)	3.9 ± 1.6	3.7 ± 1.0	3.7 ± 1.1
Platelets (150–350 × 10^9^/L)	196.8 ± 74.0	201.0 ± 87.3	213.0 ± 121.2
Glycemia (73–110 mg/dL)	116.7 ± 58.9	113.1 ± 39.2	94.1 ± 12.0
Creatinine (0.66–1.09 mg/dL)	0.7 ± 0.1	0.8 ± 0.2	0.7 ± 0.1
Urate (2.7–5.9 mg/dL)	4.8 ± 1.5	5.0 ± 1.1	5.0 ± 1.1
Cholesterol (< 200 mg/dL)	177.4 ± 40.5	185.5 ± 41.9	186.0 ± 53.2
Triglycerides (< 200 mg/dL)	96.1 ± 48.4	104.8 ± 54.4	98.3 ± 54.4
HDL (> 40 mg/dL)	54.7 ± 22.0	58.0 ± 23.4	65.4 ± 28.6
LDL (< 130 mg/dL)	102.0 ± 31.1	99.5 ± 38.0	110.9 ± 40.4
AST (< 35 U/L)	26.6 ± 11.0	21.3 ± 4.3	20.8 ± 4.2
ALT (< 35 U/L)	22.6 ± 9.8	21.4 ± 8.6	20.7 ± 8.6
LDH (140–280 U/L)	210.4 ± 86.0	202.5 ± 89.0	218.4 ± 75.7
GGT (< 38 U/L)	23.5 ± 21.6	21.9 ± 13.5	26.4 ± 25.7
B12 vitamin (200–900 pg/mL)	406.9 ± 149.2	449.8 ± 185.8	359.1 ± 94.0
Folate (2.7–17.0 ng/mL)	9.2 ± 7.0	13.3 ± 16.1	5.4 ± 2.3
Iron (27–151 µg/dL)	91.6 ± 29.5	92.0 ± 42.5	78.3 ± 24.2
Ferritin (males: 30–300 ng/mL females: 30–200 ng/mL)	M: 503.0 ± 470.9 F: 256.0 ± 223.0	M: 459.6 ± 415.7 F: 184.1 ± 188.3	M: 260.7 ± 117.2 F: 237.0 ± 291.5

Hematological, and biochemical laboratory test mean ± standard deviation before the switch and after 12 and 24 months with eliglustat

Table 3. Analytical results.

### Biomarkers

At the first year checkpoint, two patients were lost to follow-up due to COVID-19 lockdown.

#### Classic GD1 biomarkers

##### ChT activity

Chitotriosidase activity was null in three of the included patients. They were excluded from the analysis of this biomarker.

Three of the patients have normal levels for this biomarker before switching to eliglustat (10%), but after one year of treatment, only one patient maintained levels in the normal range, while the other two patients increased their levels. After two years of treatment, three patients returned to the normal range and three others reached it (20%; 6 patients).

**Fig. 2:**
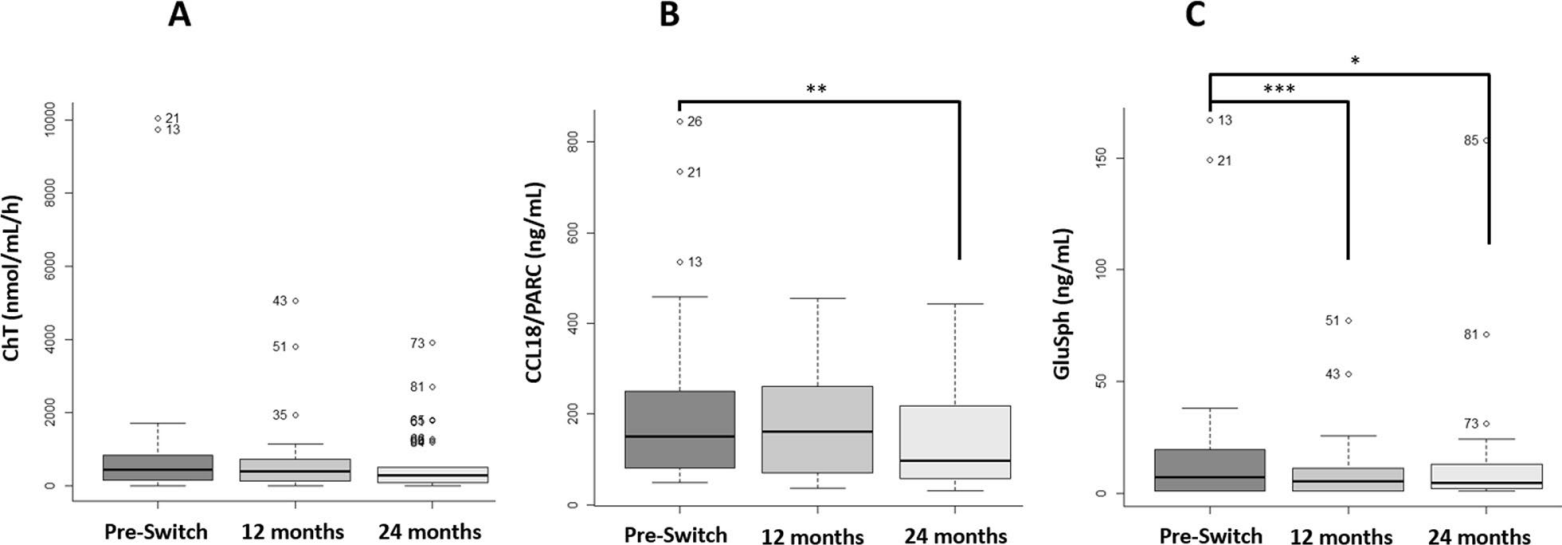
Boxplot comparing the classical biomarkers before the switch and after one and two years of treatment. Graphical representation of the mean results for the classical biomarkers before eliglustat treatment and after one and two years **A** ChT activity; **B** CCL18/PARC concentration; **C** GluSph concentration. ChT: Chitotriosidase activity; GluSph: glucosilsphingosine. *p value < 0.05; **p value < 0.01; ***p value < 0.001

After the first year of treatment with eliglustat, most of the patients reduce the ChT values (60%; 18 patients) with a reduction of about 1.1 fold, which is not statistically significant (*p* = 0.0714; Fig. 2A).

Comparing the results before the switch and after two years of treatment, the number of patients with reduced ChT activity increased slightly (63%; 19 patients), with a 1.75 fold reduction. However, this change was not statistically significant (*p* = 0.1529, Fig. 2A).

##### CCL18/PARC concentration

Most patients achieve normal levels before switching to eliglustat (57%; 17 patients). This percentage decreased after one year of treatment (50%; 15 patients) and increased again after two years of treatment (73%; 22 patients).

After one year on the new treatment, about half of the patients had a decrease in CCL18 levels (53%; 16 patients), while the other half had an increase, resulting in no change in mean levels (*p* = 0.2488; Fig. 2B).

Two years after the change, the reduction of the concentration affects more patients (83%, 25 patients) with a change on the mean values about 1.55 fold, this reduction being statistically significant when compared with the values before the change of treatment (*p* = 0.0012; Fig. 2B).

##### GluSph concentration

Before switching to eliglustat, only 9 patients had undetectable values for GluSph (30%), after one year of treatment this number increased to 25 (83%) (See details data in Additional file 2: Table S2).

Comparing the results between before the switch and the first year with the new treatment, almost all patients reduce the GluSph concentration (83%, 25 patients), with a 1.37 fold mean decrease. This decrease was higher two years after the switch, being about 1.64 fold.

The difference between the mean concentration before the switch and after one and two years was statistically significant (*p* = 0.0008 and *p* = 0.0245, respectively; Fig. 2C).

#### Newly evaluated biomarkers

To look forward, the identification and characterization of new molecules may lead to innovative biochemical diagnostic approaches and follow-up of response to therapy on GD. In this line, we have evaluated the results at baseline and in the follow-up at 12 and 24 months YKL-40, cathepsin S, hepcidine and lipocaline-2.

##### YKL-40

After one year of treatment, the levels of this chemokine were reduced by about 1.35 times, but this change is not statistically significant (*p* = 0.6819).

**Fig. 3:**
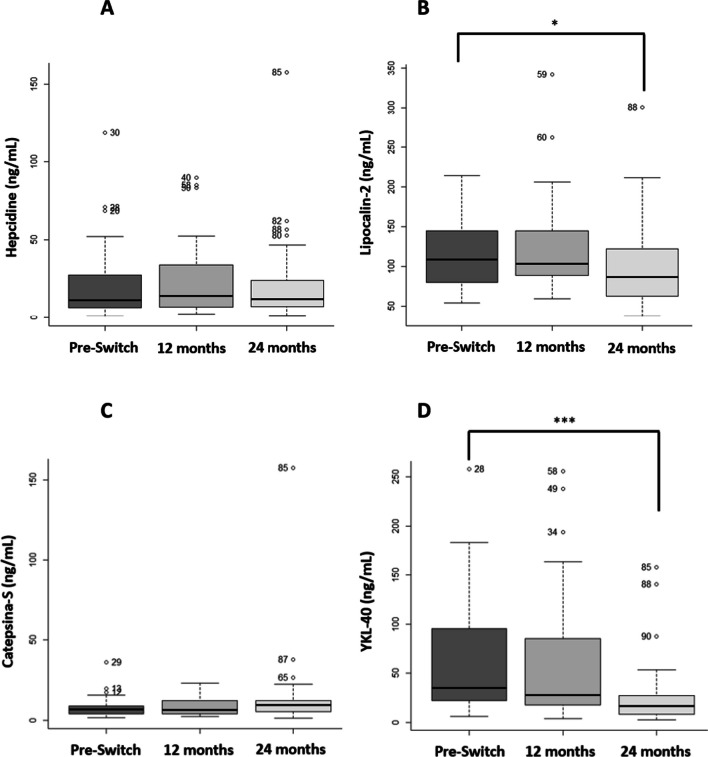
Boxplot comparing the new biomarkers before the switch and after one and two years of treatment. Graphical representation of the mean results for the new biomarkers before eliglustat treatment and after one and two years **A** Hepcidine concentration; **B** Lipocaline-2 concentration; **C** Catepsine-S concentration; D)YKL-40 concentration. *p value < 0.05; **p value < 0.01; ***p value < 0.001 and ****p value < 0.0001

Meanwhile, after two years, this change was approximately 2.35 fold with a statistically significant difference compared to the values before switching (*p* = 0.00004; Fig. 3D).

##### Cathepsin S

After one year, median cathepsin S levels were about the same as before switching to eliglustat; after two years, these median levels increased 1.35 fold, but it was not statistically significant (*p* = 0.1936 and *p* = 0.0961 for one and two years, respectively) (Fig. 3C).

##### Hepcidine

The change in median values between pre-switch and post-switch values after one year of treatment was approximately 1.27 fold, decreasing to 1.1 fold after two years of treatment. None of these differences were statistically significant (*p* = 0.0941 and *p* = 0.7496, respectively) (Fig. 3A).

##### Lipocaline-2

Differences between GD patients at baseline and healthy controls were analyzed for this new biomarker because no previous information was available, we observed an increase of lipocalin-2 in GD patients compared to controls (data not shown).

After one year of treatment with eliglustat, lipocalin-2 concentration decreased approximately 1.07 fold, and this reduction was higher after two years of treatment (approximately 1.27 fold), being statistically significant after this period (*p* = 0.3109 and *p* = 0.0155, respectively) (Fig. 3B).

#### Comparison results between patients treated previously with miglustat or ERT

**Fig. 4:**
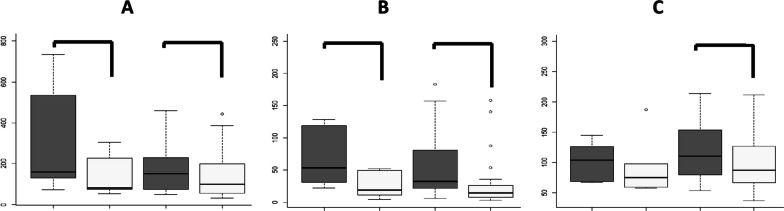
Boxplot comparing the new biomarkers before the switch and after two years of treatment in patients previously treated with miglustat or ERT. Graphical representation of the mean results for the new biomarkers before eliglustat treatment and after two years **A** CCL18/PARC concentration; **B** YKL-40 concentration; **C** Lipocalin-2 concentration. *p value < 0.05; **p value < 0.01

We have analyzed separately patients previously treated with miglustat or ERT in order to to explore whether there are differences in biomarker follow-up at two years of treatment. In spite of the scarce number of patients included in miglustat group (N = 6) we have observed after two years on eliglustat therapy a significant reduction only in CCL18/PARC (*p* = 0.031) and YKL24 levels (*p* = 0.031) while in the ERT group was also observe in Lipocalin-2 (*p* = 0.017) (Fig. 4).

## Discussion

GD1 is one of the rare diseases with several treatment options [5–8], this availability implies the need to evaluate the safety and efficacy of these treatments in real life, outside the standardized follow-up performed during clinical trials.

The availability of different treatments and the extensive screening of GD in Spain, where almost all patients are identified and treated if necessary [35, 36], is the reason why this study did not report information about patients with eliglustat as first-line treatment.

The clinical results obtained in this study after two years of treatment with eliglustat generally show stability of the measured parameters. Since these are patients with a median exposure to ERT of 17.0 years and most patients were stable in normal parameters, the extent of improvement is limited, but a finding of no worsening after switching is already important. These results are consistent with data collected in pre-approval clinical trials, such as the ENCORE study [13, 14]. In the ENGAGE study, in which eliglustat was administered as first-line therapy, improvements in some parameters such as visceral volume were observed [12].

Most patients with GD1 have bone symptoms, which are one of the most limiting factors in their daily lives. In the ENGAGE study, improvements in bone and bone marrow disease indicators were observed, while in the ENCORE study the values remained stable [12–14]. In addition, these real-world studies have shown stability in bone symptoms [15–17], but the results of our study did not show any changes in bone mineral density or T and Z scores after switching treatment, probably because of disease stabilization with previous treatment or the short duration of the study. It is important to consider the gender and age of patients as physiological factors that may affect the loss of bone mineral density.

Regarding health-related quality of life, it was maintained from the switch throughout treatment for one and two years in oral therapy patients, which may be attributed to the low severity of treatment-related adverse effects, most of them gastrointestinal symptoms. In contrast to other publications with discontinuation rates of 10–37%, none of the patients in our cohort required discontinuation [18, 37]. In addition, none of our patients, as well as none of the patients of Istaiti et al. reported cardiac AEs, probably due to the extensive pre-treatment evaluation [37].

A significant increase was observed in physical function scores and in the reduction of bodily pain, with scores close to those of the general Spanish population. The mental health area also shows improvement, although it is not significant, but we consider that the study was conducted during the coronavirus pandemic and this point may be influenced by this circumstance. The change in drug administration may also be reflected in these results due to the gain of comfort and time availability with the oral therapy.

The discrepancies observed between the information obtained in the medical desk about pain and fatigue with the information provided by the patients in the quality of life surveys supports the physician's and patient's different perceptions of these symptoms and their evaluation. The incorporation of PROMs indicators for understanding the impact of GD on quality of life and patient’s perceptions on care, can guide in decision-making processes [38].

Regarding laboratory tests, the limitation is the same as before, as most of the values are within the normal range due to the previous treatment. As before, the ENGAGE study shows an improvement in hemoglobin levels and platelet counts when the treatment is administered as first line [12]. Ferritin is one of the biomarkers that did not follow this trend, obtaining values highly increased with the previous treatment in both sexes and, although the values were reduced after the change they did not reach the normal range in females and did it but in the higher range in males. The interpretation of this marker is difficult because of the limited number of patients and the unspecified of this inflammatory maker, involved in other underlying processes. This trend has been previously observed in patients treated with ERT, where levels decreased after treatment but remained above normal levels [26].

The important role of biomarkers in GD has been previously described both at diagnosis and for follow-up during therapy [19]. Biomarker analysis showed, as in daily practice, a maintained stability of levels one and two years after treatment change. After 2 years and despite the effect of the previous treatment, the reduction of CCL18/PARC and GluSph was statistically significant. This result may be related to the stimulation after switching and is in agreement with those previously reported [18, 37, 39].

Regarding other biomarkers not as well established as the previous ones, YKL-40, known as chitinase 3-like-1, has previously been shown to have increased expression in GD patients and has been associated with disease activity in the compartment with low ERT response [25]. This biomarker has been associated with the musculoskeletal system and increased in malignant bone diseases [40, 41]. Following this thinking, the results we obtained could be an early identification of bone improvement that needs to be confirmed with extended follow-up.

In addition, lipocalin-2, previously described as a possible biomarker of GD [42], showed a significant improvement after eliglustat treatment. The decrease in levels may be due to the relationship between lipocalin-2 and inflammatory mechanisms [43]. It has been shown that this molecule is activated by proinflammatory cytokines, which are altered in patients with Gaucher disease compared to controls [44]. This reduction has been more evident in patients previously treated with ERT.

In conclusion, in our study, treatment with eliglustat maintains the efficacy achieved with ERT in adult patients with GD1 in real life, both in clinical practice and in laboratory testing. In addition, a reduction in levels of classical biomarkers was achieved after switching, as well as a reduction in new biomarkers such as lipocalin-2 and YKL-40. Finally, the adverse events are not severe enough to require the previous treatment to be restarted.

## Limitation of study

The small number of patients is always one of the most important limitations of studies focused on rare diseases. In this case, it is also possible that we are limited by a selection bias regarding the clinical stability of the disease in the patients, since most of them had achieved therapeutic goals with ERT and had been treated for a long time (about 17.0 years).

### Supplementary Information

**Additional file 1. Table S1**. Inclusion and exclusion criteria.

**Additional file 2. Table S2**. Results of biomarkers determination at baseline, after 12 and 24 months on therapy.

## Data Availability

The data base and materials of study are deposited with the FEETEG and available upon request from the corresponding author.
