# Supplementary material for: Real life data: follow-up assessment on Spanish Gaucher disease patients treated with eliglustat. TRAZELGA project
Source: Orphanet J Rare Dis. 2023 Dec 15;18:390. doi: 10.1186/s13023-023-02939-4 (PMC10722815; doi:10.1186/s13023-023-02939-4)
Supplement: Supplementary file 1 — Additional file 1. Table S1. Inclusion and exclusion criteria. [file 13023_2023_2939_MOESM1_ESM.docx]

Table 1S.-Patients participating in the study will be included consecutively and must meet all of the following selection criteria meet all of the following selection criteria:

Inclusion criteria

1.- CYP2D6 metabolizers: fast, intermediate or slow, without previous therapy with eliglustat, symptomatic of both sexes and over 18 years of age, diagnosed according to accepted criteria for Gaucher disease type 1 *.

2.- No history of cardiac disorders, that predisposing to arrhythmias, recent myocardial infarction, ventricular arrhythmia, long QT síndrome, bradycardia, heart block and

congestive heart failure and combination with class IA treated with antiarrhythmics. (e.g. quinidine) or Class III (e.g. amiodarone, sotalol).

3.- All patients must sign the Informed Consent Form.

4.- According to SmPC it is preferable to avoid the use of Cerdelga during pregnancy.

Therefore, it is advisable to use reliable contraceptives during the study.

Exclusion criteria

1.- Patients with type 2 or type 3 Gaucher disease.

2.- Pregnant or lactating women.

3.- Severe renal or hepatic dysfunction.

4.- Vertigo or central nervous system involvement.

5.- Current participation in a clinical trial, or previous (within the last four weeks) or concomitant

administration of any other experimental drug.

6.- Cognitive or mental disorders that preclude adequate assessment of symptomatic response according to clinical judgment of the investigator.
