# Supplementary material for: Real life data: follow-up assessment on Spanish Gaucher disease patients treated with eliglustat. TRAZELGA project
Source: Orphanet J Rare Dis. 2023 Dec 15;18:390. doi: 10.1186/s13023-023-02939-4 (PMC10722815; doi:10.1186/s13023-023-02939-4)
Supplement: Supplementary file 2 — Additional file 2. Table S2. Results of biomarkers determination at baseline, after 12 and 24 months on therapy. [file 13023_2023_2939_MOESM2_ESM.docx]

| **Patients** | **Quitotriosidase** | | | **CCL18/PARC** | | | **Glucosylsphingosine** | | |
| --- | --- | --- | --- | --- | --- | --- | --- | --- | --- |
|  | **Pre-switch** | **12 months** | **24 months** | **Pre-switch** | **12 months** | **24 months** | **Pre-switch** | **12 months** | **24 months** |
| 1 | 21.40 | 24.20 | 3.05 | 148.00 | 258.00 | 232.00 | 19.66 | 7.47 | 24.18 |
| 2 | 51.70 | 44.40 | 10.21 | 171.00 | 316.00 | 94.00 | 5.30 | <0.88 | 2.21 |
| 3 | 183.20 | 13.69 | 2.64 | 81.00 | 98.00 | 58.00 | <0.88 | 2.10 | 2.45 |
| 4 | 151.10 | 193.87 | 24.50 | 251.00 | 118.00 | 101.00 | 6.09 | 5.50 | 4.94 |
| 5 | 43.70 | 18.74 | 15.99 | 161.00 | 266.00 | 231.00 | 8.38 | 7.93 | 10.68 |
| 6 | 33.00 | 38.70 | 12.84 | 63.00 | 63.00 | 85.00 | <0.88 | 7.24 | 6.09 |
| 7 | 18.50 | 5.14 | 4.96 | 314.00 | 250.00 | 221.00 | 18.83 | 10.21 | 4.63 |
| 8 | 123.70 | 15.30 | 3.13 | 110.00 | 173.00 | 130.00 | 19.60 | 19.28 | 13.13 |
| 9 | 53.70 | 65.30 | 19.65 | 57.00 | 76.00 | 51.00 | 7.52 | <0.88 | 2.60 |
| 10 | 21.70 | 13.60 | 10.47 | 133.00 | 97.00 | 48.00 | <0.88 | <0.88 | 0.97 |
| 11 | 31.00 | 42.70 | 17.27 | 50.00 | 68.00 | 43.00 | 5.18 | <0.88 | <0.88 |
| 12 | 66.64 | 22.76 | 10.05 | 123.00 | 73.00 | 69.00 | 32.20 | 25.67 | 16.34 |
| 13 | 37.10 | 32.40 | 4.28 | 535.00 | 286.00 | 227.00 | 167.10 | 53.39 | 31.25 |
| 14 | 69.80 | 70.50 | 49.39 | 72.00 | 65.00 | 52.00 | <0.88 | <0.88 | 3.01 |
| 15 | 128.20 | 163.70 | 11.04 | 140.00 | 92.00 | 82.00 | <0.88 | <0.88 | 2.14 |
| 16 | 23.20 | 142.40 | 7.81 | 69.00 | 41.00 | 31.00 | 9.30 | 5.34 | 5.09 |
| 17 | 12.30 | 12.50 | 8.18 | 69.00 | 46.00 | 56.00 | <0.88 | <0.88 | <0.88 |
| 18 | 95.20 | 28.10 | 23.36 | 68.00 | 37.00 | 47.00 | <0.88 | 1.62 | 3.76 |
| 19 | 118.60 | 237.80 | 51.98 | 131.00 | 205.00 | 75.00 | 5.31 | <0.88 | 1.56 |
| 20 | 22.16 | 21.98 | 17.52 | 180.00 | 200.00 | 79.00 | 12.34 | 6.70 | 4.18 |
| 21 | 31.00 | 28.20 | 20.91 | 734.00 | 455.00 | 304.00 | 149.02 | 77.20 | 71.15 |
| 22 | 48.30 | 99.80 | 35.75 | 209.00 | 240.00 | 144.00 | 6.60 | <0.88 | 3.20 |
| 23 | 23.90 | - | 12.94 | 201.00 | - | 98.00 | 13.40 | - | 7.50 |
| 24 | 25.60 | 25.39 | 6.45 | 152.00 | 170.00 | 101.00 | 21.10 | 11.79 | 21.19 |
| 25 | 32.10 | - | 157.80 | 82.00 | - | 157.80 | <0.88 | - | 157.80 |
| 26 | 156.70 | 126.60 | 53.47 | 846.00 | 387.00 | 443.00 | 22.70 | 7.15 | 6.96 |
| 27 | 6.09 | 3.82 | 27.54 | 439.00 | 391.00 | 385.00 | 37.90 | 19.70 | 20.61 |
| 28 | 258.10 | 255.70 | 140.88 | 197.00 | 283.00 | 218.00 | <0.88 | <0.88 | 1.50 |
| 29 | 12.43 | 18.77 | 17.97 | 262.00 | 52.00 | 98.00 | 27.65 | 12.87 | 6.39 |
| 30 | 20.59 | 16.53 | 87.52 | 459.00 | 154.00 | 178.00 | 1.38 | 3.80 | 1.95 |
| **Median**  **(Q1-Q3)** | 435.5  (158.25-830.75) | 400.3  (134.12-811.05) | 281.2  (98.2-683.67) | 150.0  (78.75-253.75) | 162.0  (69.25-264.00) | 98.0  (57.50-218.75) | 7.1  (0.88-20.02) | 5.4  (0.88-11.40) | 4.8  (2.19-13.93) |

| **Patients** | **Cathepsine-S** | | | **Lipocalin-2** | | | **Hepcidine** | | |
| --- | --- | --- | --- | --- | --- | --- | --- | --- | --- |
|  | **Pre-switch** | **12 months** | **24 months** | **Pre-switch** | **12 months** | **24 months** | **Pre-switch** | **12 months** | **24 months** |
| 1 | 7.29 | 15.07 | 12.81 | 61.00 | 70.00 | 40.49 | 6.53 | 8.22 | 5.60 |
| 2 | 7.13 | 6.15 | 3.57 | 204.00 | 101.00 | 81.36 | 10.18 | 8.09 | 3.63 |
| 3 | 3.87 | 4.16 | 9.81 | 78.00 | 90.00 | 130.68 | 5.70 | 17.42 | 10.53 |
| 4 | 5.30 | 5.33 | 2.84 | 142.00 | 110.00 | 83.99 | 19.31 | 6.95 | 12.20 |
| 5 | 7.13 | 12.43 | 26.68 | 213.00 | 84.49 | 152.38 | 8.55 | 22.20 | 3.53 |
| 6 | 4.06 | 6.11 | 7.74 | 143.00 | 152.00 | 117.55 | 4.61 | 10.08 | 9.52 |
| 7 | 4.74 | 3.12 | 1.14 | 101.00 | 87.00 | 37.11 | 35.83 | 30.27 | 46.70 |
| 8 | 4.73 | 7.68 | 16.47 | 153.00 | 206.00 | 134.09 | 10.89 | 4.54 | 9.45 |
| 9 | 3.10 | 3.11 | 10.65 | 85.00 | 143.00 | 99.43 | 1.77 | 2.73 | 2.66 |
| 10 | 6.72 | 3.43 | 1.20 | 201.00 | 98.00 | 111.99 | 40.22 | 89.84 | 22.69 |
| 11 | 1.63 | 2.39 | 6.16 | 54.00 | 85.00 | 61.36 | 4.03 | 7.81 | - |
| 12 | 17.42 | 6.84 | 6.32 | 79.00 | 75.18 | 47.31 | 10.85 | 8.05 | 22.87 |
| 13 | 19.64 | 23.26 | 16.53 | 145.00 | 197.00 | 86.49 | 7.14 | 27.26 | 23.65 |
| 14 | 2.73 | 8.29 | 8.88 | 69.00 | 91.00 | 97.80 | 11.68 | 34.02 | - |
| 15 | 14.65 | 21.63 | 5.25 | 104.00 | 108.00 | 59.36 | 5.21 | 6.93 | 7.62 |
| 16 | 8.85 | 15.05 | 4.73 | 154.00 | 169.00 | 74.62 | 11.84 | 11.26 | 11.77 |
| 17 | 7.02 | 5.15 | 1.89 | 77.00 | 102.00 | 88.55 | 6.59 | 2.22 | 16.59 |
| 18 | 4.91 | 7.72 | 1.87 | 113.00 | 105.00 | 72.05 | 19.21 | 34.68 | 24.45 |
| 19 | 3.61 | 5.74 | 9.11 | 104.00 | 142.00 | 58.18 | 19.93 | 16.50 | 11.23 |
| 20 | 3.33 | 8.67 | 11.91 | 126.00 | 75.36 | 63.69 | 68.56 | 83.28 | 52.72 |
| 21 | 15.48 | 12.09 | 12.32 | 67.00 | 101.00 | 187.36 | 0.86 | 3.22 | 1.39 |
| 22 | 6.79 | 3.86 | 10.44 | 96.00 | 114.00 | 53.86 | 51.88 | 49.26 | 62.13 |
| 23 | 7.66 | - | 5.79 | 80.00 | - | 86.23 | 27.46 | - | 6.84 |
| 24 | 5.97 | 14.68 | 22.44 | 111.00 | 91.00 | 71.05 | 24.23 | 2.56 | 8.54 |
| 25 | 8.91 | - | 157.80 | 197.00 | - | 157.80 | 10.12 | - | 157.80 |
| 26 | 3.43 | 4.03 | 10.28 | 80.00 | 104.00 | 91.86 | 45.16 | 52.55 | 28.86 |
| 27 | 8.85 | 4.00 | 37.92 | 108.00 | 146.00 | 122.05 | 11.53 | 26.53 | 19.45 |
| 28 | 3.22 | 3.98 | 11.21 | 125.00 | 59.00 | 301.06 | 70.73 | 84.94 | 56.58 |
| 29 | 36.27 | 13.18 | 5.55 | 109.62 | 342.42 | 62.46 | 4.74 | - | 3.46 |
| 30 | 9.74 | 12.12 | 10.55 | 214.00 | 262.20 | 211.31 | 118.70 | - | - |
| **Median**  **(Q1-Q3)** | 6.8  (3.80-8.87) | 6.5  (4.01-12.35) | 9.5  (5.12-12.44) | 108.8  (79.75-147.00) | 103.0  (87.75-145.25) | 86.4  (62.19-124.21) | 11.2  (6.32-29.55) | 13.9  (6.95-34.19) | 11.8  (6.84-24.45) |

| **Patients** | **YKL-40** | | |
| --- | --- | --- | --- |
|  | **Pre-switch** | **12 months** | **24 months** |
| 1 | 21.40 | 24.20 | 3.05 |
| 2 | 51.70 | 44.40 | 10.21 |
| 3 | 183.20 | 13.69 | 2.64 |
| 4 | 151.10 | 193.87 | 24.50 |
| 5 | 43.70 | 18.74 | 15.99 |
| 6 | 33.00 | 38.70 | 12.84 |
| 7 | 18.50 | 5.14 | 4.96 |
| 8 | 123.70 | 15.30 | 3.13 |
| 9 | 53.70 | 65.30 | 19.65 |
| 10 | 21.70 | 13.60 | 10.47 |
| 11 | 31.00 | 42.70 | 17.27 |
| 12 | 66.64 | 22.76 | 10.05 |
| 13 | 37.10 | 32.40 | 4.28 |
| 14 | 69.80 | 70.50 | 49.39 |
| 15 | 128.20 | 163.70 | 11.04 |
| 16 | 23.20 | 142.40 | 7.81 |
| 17 | 12.30 | 12.50 | 8.18 |
| 18 | 95.20 | 28.10 | 23.36 |
| 19 | 118.60 | 237.80 | 51.98 |
| 20 | 22.16 | 21.98 | 17.52 |
| 21 | 31.00 | 28.20 | 20.91 |
| 22 | 48.30 | 99.80 | 35.75 |
| 23 | 23.90 | - | 12.94 |
| 24 | 25.60 | 25.39 | 6.45 |
| 25 | 32.10 | - | 157.80 |
| 26 | 156.70 | 126.60 | 53.47 |
| 27 | 6.09 | 3.82 | 27.54 |
| 28 | 258.10 | 255.70 | 140.88 |
| 29 | 12.43 | 18.77 | 17.97 |
| 30 | 20.59 | 16.53 | 87.52 |
| **Median**  **(Q1-Q3)** | 35.1  (22.04-101.05) | 28.2  (17.08-92.48) | 16.6  (8.08-29.59) |
